# Rising Prevalence of Low-Frequency *PPM1D* Gene Mutations after Second HDCT in Multiple Myeloma

**DOI:** 10.3390/cimb46080484

**Published:** 2024-07-29

**Authors:** Katja Seipel, Nuria Z. Veglio, Henning Nilius, Barbara Jeker, Ulrike Bacher, Thomas Pabst

**Affiliations:** 1 Department for Biomedical Research, University of Bern, 3008 Bern, Switzerland; 2 Department of Medical Oncology, University Hospital Bern, 3010 Bern, Switzerland; nuria.veglio@students.unibe.ch (N.Z.V.); barbara.jeker@insel.ch (B.J.); 3 Department of Clinical Chemistry, University of Bern, 3010 Bern, Switzerland; henning.nilius@insel.ch; 4 Department of Hematology, University Hospital Bern, 3010 Bern, Switzerland; veraulrike.bacher@insel.ch

**Keywords:** autologous stem cell transplant (ASCT), clonal hematopoiesis (CH), multiple myeloma (MM), protein phosphatase Mg/Mn-dependent 1D (PPM1D), wild-type p53-induced phosphatase 1 (Wip1), next-generation sequencing (NGS)

## Abstract

Multiple myeloma (MM) first-line treatment algorithms include immuno-chemotherapy (ICT) induction, high-dose chemotherapy (HDCT) and autologous stem cell transplant (ASCT) consolidation, followed by lenalidomide maintenance. After these initial therapies, most patients suffer a disease relapse and require subsequent treatment lines including ICT, additional HDCT and ASCT, or novel immunotherapies. The presence of somatic mutations in peripheral blood cells has been associated with adverse outcomes in a variety of hematological malignancies. Nonsense and frameshift mutations in the *PPM1D* gene, a frequent driver alteration in clonal hematopoiesis (CH), lead to the gain-of-function of Wip1 phosphatase, which may impair the p53-dependent G1 checkpoint and promote cell proliferation. Here, we determined the presence of *PPM1D* gene mutations in peripheral blood cells of 75 subsequent myeloma patients in remission after first or second HDCT/ASCT. The prevalence of truncating *PPM1D* gene mutations emerged at 1.3% after first HDCT/ASCT, and 7.3% after second HDCT/ASCT, with variant allele frequencies (VAF) of 0.01 to 0.05. Clinical outcomes were inferior in the *PPM1D*-mutated (*PPM1D*mut) subset with median progression-free survival (PFS) of 15 vs. 37 months (*p* = 0.0002) and median overall survival (OS) of 36 vs. 156 months (*p* = 0.001) for the *PPM1D*mut and *PPM1D*wt population, respectively. Our data suggest that the occurrence of *PPM1D* gene mutations in peripheral blood cells correlates with inferior outcomes after ASCT in patients with multiple myeloma.

## 1. Introduction

Multiple myeloma (MM) is a neoplasm of clonal plasma cells and accounts for approximately 10% of all hematological malignancies [1,2]. Standard first-line treatment options include immuno-chemotherapy (ICT), high-dose chemotherapy (HDCT), autologous stem cell transplant (ASCT) and long-term immunomodulatory drug (IMiD) maintenance [3]. Remission may be induced by initial therapy with three to four components (triplet or quadruplet therapy), including monoclonal antibodies (daratumumab), immunomodulatory drugs (lenalidomide), proteasome inhibitors (bortezomib) and steroids (dexamethasone) [4,5]. The introduction of immunomodulatory drugs, proteasome inhibitors, and monoclonal antibodies has improved the treatment outcome to a five-year survival rate of 50–58% [6]. After first-line therapy, 20% to 50% of myeloma patients undergo stem cell mobilization chemotherapy, high-dose chemotherapy (HDCT) and autologous stem cell transplantation (ASCT). HDCT consists of alkylating agents including melphalan, bendamustine and treosulfan [7,8]. HDCT and ASCT are standard therapies in young and middle-aged patients (below the age of 60), and can be offered to older patients without therapy-limiting comorbidities. HDCT/ASCT can provide a deep response with longer time to next treatment, but is associated with significant toxicities including cytopenias. Moreover, high-dose melphalan exposure increases both the overall and nonsynonymous mutational burden detected between myeloma diagnosis and relapse by ~10–20% [9]. A better understanding of which patients may benefit from HDCT/ASCT and maintenance therapy is an important question and active area of ongoing clinical investigation.

Clonal hematopoiesis (CH) refers to an expansion of clonally derived hematopoietic cells with gene mutations conferring a selective advantage to hematopoietic stem cells [10]. The most relevant CH driver genes include *PPM1D*, *TP53*, *DNMT3A*, *ASXL1* and *TET2*, but more driver genes have been detected recently [11]. The *PPM1D* gene is located on chromosome 17q and encodes the protein phosphatase Mg2+/Mn2+-dependent 1D (PPM1D, Wip1), which can dephosphorylate the tumor suppressor protein p53 and other proteins involved in the DNA damage response (DDR) [12]. *PPM1D* gene alterations entail nonsense and frameshift mutations, creating early stop codons [13,14]. The truncated PPM1D protein products may dephosphorylate p53, but are not recognized by the APC/C complex, leading to the stabilization and accumulation of a constitutively active protein phosphatase. In the general population, 0.13–5% carry a mutation in the *PPM1D* gene across all age groups, with an increase in somatic mutations with age [12,13]. More than 30% of patients with lymphoma who had undergone HDCT/ASCT were found to have CH, and *PPM1D* was the most frequent driver alteration [15,16]. Clonal hematopoiesis was associated with adverse outcomes after ASCT in lymphoma [15] and MM [17]. According to the MMRF COMMPASS study, the CHIP mutation prevalence rises from 5.8% at MM diagnosis to 25% 3 years after ASCT. Moreover, the presence of *DNMT3A* and *PPM1D* mutations in stem cell grafts collected in myeloma patients correlated with reduced stem cell yields and delayed platelet count recovery following ASCT associated with impaired regeneration in ASCT recipients [18].

In this retrospective study, we determined the prevalence of *PPM1D* gene mutations in MM patients who had undergone one or two HDCT/ASCT procedures and analyzed the impact of *PPM1D* mutations on outcome. The presence of mutations in *PPM1D* exon 6 was assessed through NGS amplicon sequencing of genomic DNA extracted from peripheral blood mononuclear cells (PBMC). We analyzed the correlations between *PPM1D* mutational status and clinical outcomes after ASCT, including the response rates and survival times. Low-frequency *PPM1D* gene mutations may affect treatment response to ASCT in multiple myeloma.

## 2. Materials and Methods

### 2.1. Patient Samples

We conducted a retrospective single-center study at the Inselspital, University Hospital Bern, Switzerland. The cohort comprised 75 patients diagnosed with multiple myeloma (MM), who were treated with first-line therapy, HDCT and autologous stem cell transplant (ASCT) between June 2003 and June 2022. Patients with disease relapse received up to seven therapy lines and up to four HDCT/ASCT therapies. All participants signed the informed consent document for the usage of personal data for research purposes. All patients were followed up clinically at one, three, six months and annually after ASCT. In addition, the clinical and laboratory data related to the underlying disease, the ASCT and survival endpoints were systematically collected.

### 2.2. NGS Amplicon Sequencing

Genomic DNA was extracted from mononuclear cells (PBMCs) isolated from the peripheral blood of 80 MM patients collected after autologous stem cell transplant and 8 healthy donors. NGS amplicon sequencing and bioinformatics analysis were performed at Microsynth, Balgach, Switzerland. NGS amplicon sequencing included the preparation of a Nextera two-step PCR library, sequencing on Illumina MiSeq, 2 X 300 bases using a MiSeq Reagent Kit v3 (600 cycle) and gene-specific primers covering exon 6 of the *PPM1D* gene (F: 5′-GA GGATCCATGGCCAAGGG-3′, R: 5′-TTCCAATTTTCTTCTGGCCCC-3′, product size: 505 bp). The bioinformatic analysis included the trimming of locus-specific Illumina adapter primers; merging of the reads; mapping the trimmed and merged reads to the human reference chromosome 17 for variant calling and annotation, and to the PPM1D-selected region (chr17:60,662,994–60,663,549) for coverage analysis; and dereplication of the trimmed and merged reads. The total number of demultiplexed reads which passed Illumina’s chastity filter was 2,132,878, and the number of demultiplexed bases was 638,516,106, with a mean read length of 299 bp. The quality of the reads was checked in fastq format with FastQC (version 0.11.9). The raw reads shorter than 200 bases, with average Q-values below 24 or incorporating un-called ‘N’ bases were filtered using the BBTools software suite (version 38.96). The quality assessment returned a mean Q of 34, with 94% in Q20 and 84% in Q30. The mapping software bwa (version 0.7.17-r1198) in combination with samtools (version 1.15.1) was used to map the remaining reads to (selected regions of) the human UCSC hg38 reference genome downloaded from iGenomes. The coverage analysis was performed with bedtools (version 2.30.0). Variant calling was performed with LoFreq software (version 2.1.5). The consequences of called variants were annotated on the amino acid level using the annotation of the mentioned reference genome. Disclaimer: DNA library construction, sequencing, and data analysis described in this section were performed at Microsynth AG (Balgach, Switzerland).

### 2.3. Clinical Data Analysis

The clinical parameters investigated for their potential prognostic significance included patient age, international prognostic index (IPI), cytogenetic risk, blood parameters, number of treatment lines, remission status before and after therapy. The progression-free survival (PFS) and overall survival (OS) were defined as the time from start of 1st immuno-chemotherapy to disease progression, death, or last follow-up, respectively. PFS and OS were censored at the last follow-up on 18 February 2024, which was also used as the data cutoff. The survival curves (Kaplan–Meyer) and univariate statistical analyses were performed on GraphPad Prism version 10 (GraphPad Software, San Diego, CA, USA). The categorical variables were summarized as frequencies and percentages, and the continuous variables as median and ranges. To test the association between *PPM1D* gene mutations and survival, univariable and multivariable Cox proportional hazards models were fitted to the data using the Survival package for R (version 4.3.1). The multivariable model was adjusted for patient age (< median age, > median age), initial stage, and cytogenetic risk group.

## 3. Results

### 3.1. Prevalence of PPM1D Mutations in Multiple Myeloma Patients after ASCT

**Figure 1:**
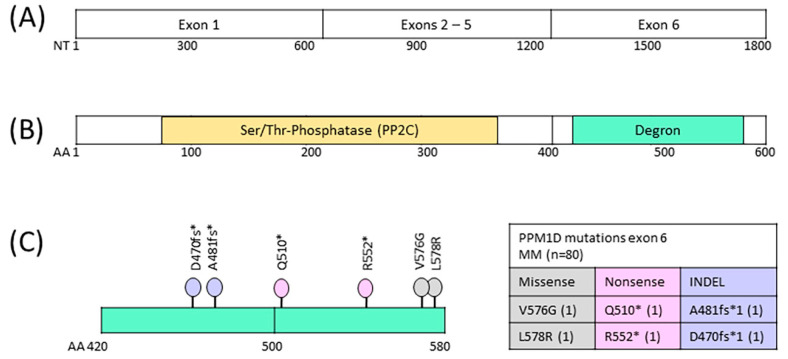
*PPM1D* gene and encoded Wip1 protein phosphatase. (**A**) *PPM1D* gene coding region structure. (**B**) PPM1D protein phosphatase (Wip1) with central phosphatase domain and C-terminal degron region. (**C**) Degron region with lollipop plot indicating positions of six identified somatic mutations. NT: nucleotide; AA: amino acid. * (asterisk) = translation termination (stop) codon.

NGS amplicon sequencing was performed to identify mutations in exon 6 of the PPM1D gene in peripheral blood mononuclear cells isolated from 75 patients diagnosed with multiple myeloma who had been treated with first-line therapy, high-dose chemotherapy (HDCT) and autologous stem cell transplant (ASCT). A total of 34 patients had one HDCT/ASCT therapy, 41 patients had more than one HDCT/ASCT procedure. Blood samples were collected after first and second ASCT. We identified six low-frequency *PPM1D* gene mutations in the peripheral blood cells of four MM patients (4/75, 5.3%), with two missense, two indel and two nonsense mutations (Table 1, Figure 1). One stop-gain and two missense mutations were detected in one sample after first ASCT, and three stop-gain mutations were detected in three samples after the second ASCT. The prevalence of truncating *PPM1D* gene mutations emerged at 1.3% (1/75) after first ASCT and 7.3% (3/41) after second ASCT. The VAF of PPM1D gene mutations called in the DNA samples ranged from 0.01 to 0.05. All four variants with stop-gain changes are translated into phosphatase protein variants lacking the APC/C (DBOX) degron motif (AA550-557) and may not be effectively targeted for degradation by the cellular APC/C complex. Consequently, the function of the p53 tumor suppressor may be impaired in cells with mutated PPM1D genes, which may promote cell survival and proliferation.

### 3.2. Clinical Characteristics of Myeloma Patients

The cohort comprised 75 patients diagnosed with multiple myeloma at a median age of 59 years (Table 2). The majority of patients were characterized by male gender, ISS II, standard cytogenetic risk, positive for paraprotein IgG and light chain kappa, normal hemoglobin, calcium and LDH levels, as well as high bone marrow infiltration rates. Half of the patients had elevated beta-2-microglobulin level indicating elevated tumor burden. Half of the patients had subnormal serum albumin levels indicating kidney disease or inflammatory disease, 20% of the patients had renal dysfunction, and 44% of patients were anemic (Hb < 110 g/L). Clinical data were correlated to *PPM1D* gene mutation status after HDCT/ASCT. While the majority of *PPM1D*wt patients were initially staged at ISS I or II, with standard cytogenetic risk, and positive for paraprotein IgG and light chain kappa, the majority of *PPM1D*mut patients were initially staged at ISS III, with standard cytogenetic risk, and positive for paraprotein IgA. The *PPM1D* gene is located on chromosome 17q, but the presence of PPM1D gene mutations was not associated with the deletion of chromosome 17p. There was, however, an elevated prevalence of cytogenetic aberration +1q in the *PPM1D*mut subset.

### 3.3. Therapeutic Interventions and Clinical Responses in Myeloma Patients

All myeloma patients were treated with bortezomib and dexamethasone in combination with lenalidomide or cyclophosphamide in first-line immuno-chemotherapy (ICT). The majority of patients (75%) had partial remission after first-line therapy (Table 3). Autologous stem cells were mobilized with G-CSF and a non-myelosuppressive mobilization chemotherapy with either vinorelbine, gemcitabine or plerixafor [19]. The CD34+ stem cell yields were adequate in the majority of myeloma patients with a median 10.7 × 10^6^ cells/kg body weight. After stem cell collection, all myeloma patients underwent high dose chemotherapy (HDCT) and autologous stem cell transplant (ASCT). HDCTs consisted of alkylating agent melphalan and, more recently, melphalan in combination with bendamustine or treosulfan [7,8]. After HDCT, the ASCT recipients were infused with a median number of 3.8 × 10^6^ CD34+ cells/kg. The ASCT recipients received a median three adult platelet doses in the post-transplant period, with median nine platelet doses in the *PPM1D*mut subgroup. The majority of patients (64%) had complete remission after first ASCT. Relapses occurred in the majority of patients (72%), which then required subsequent ICT lines and HDCT/ASCT procedures. The second-line ICT consisted of dexamethasone in combination with lenalidomide, carfilzomib, daratumumab, isatuximab or elotuzumab [20]. Again, the majority of patients (27/52, 52%) had partial remission after the second-line ICT. The majority of patients (63%) received one or two ICT lines and one or two HDCT/ASCT, with a maximum of seven ICT lines in four patients and four HDCT/ASCT procedures in one patient. A total of 12 patients with high-risk cytogenetics received a tandem ASCT, i.e., the second HDCT/ASCT within 4 months after the first ASCT [21]; 28 patients received radiation therapy to treat plasmacytoma lesions or to palliate local symptoms due to bone or extramedullary lesions [22]. The number of ICT and HDCT/ASCT lines varied in the two subgroups. While the *PPM1D*wt subgroup received a median two ICT lines and two HDCT/ASCT lines, the *PPM1D*mut subgroup received a median three ICT lines and 2.5 HDCT/ASCT lines.

### 3.4. Inferior Clinical Outcome in Myeloma Patients with Low-Frequency PPM1D Mutations

**Figure 2:**
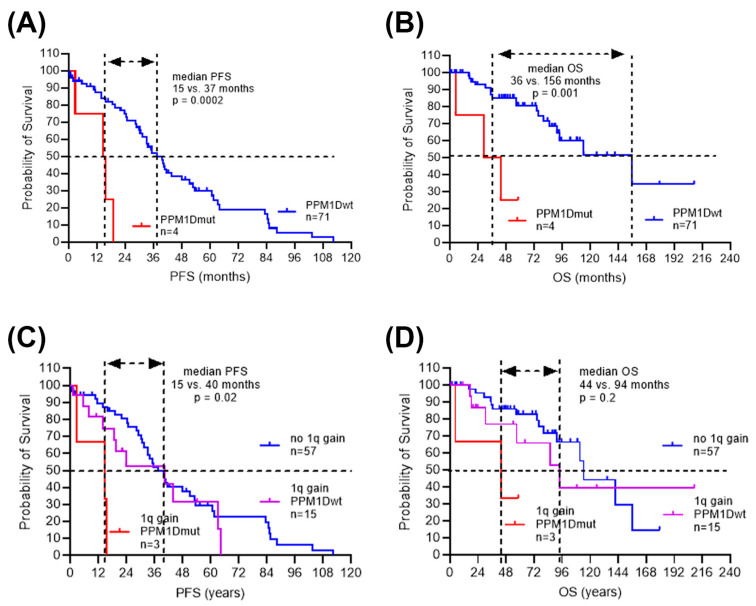
Clinical outcome in MM patients treated with ICT, HDCT and ASCT. Survival times from start of 1st ICT until 1st relapse/progression (PFS) (**A**,**C**), and survival times from start of 1st ICT until death or last follow-up date (OS) (**B**,**D**) were analyzed for MM patients stratified by *PPM1D* gene status (**A**,**B**) and by chromosome 1 status (**C**,**D**).

The survival time from initial diagnosis to disease progression and death varied significantly in the *PPM1D* wild-type versus *PPM1D*-mutated myeloma patients. In the *PPM1D* wild-type subgroup (*n* = 71), disease progressed in 49 patients (69%) with a median progression-free survival of 37 months after first ICT, and 19 patients (27%) died with a median survival of 156 months after first ICT. In the *PPM1D*-mutated subgroup (*n* = 4), disease progressed in all patients (100%) with a median progression-free survival of 15 months, and death occurred in three patients (75%) with a median survival of 36 months after first ASCT. Survival times were significantly reduced in *PPM1D*mut population (PFS median 15 vs. 37 months, *p* = 0.0002; OS median 36 vs. 156 months, *p* = 0.001) (Figure 2A,B). As there was a potential association of *PPM1D* gene mutation with cytogenetic aberration +1q, the survival times were analyzed in patients stratified according to chromosome 1q status. There was no significant difference in the median survival times in patients with chromosome 1q gain compared to normal chromosome 1. However, within the myeloma patients with 1q gain, median survival time was reduced in the *PPM1D*mut population compared to *PPM1D*wt (PFS 15 vs. 40 months, *p* = 0.02; OS 44 vs. 94 months, *p* = 0.2) (Figure 2C,D).

To account for covariance and correlation in the data, multivariate testing was performed. The multivariable model was adjusted for patient age, initial stage and cytogenetic risk group. The multivariate analysis supported the association observed in the univariate analysis for inferior clinical outcomes in myeloma patients carrying *PPM1D* gene mutations treated with HDCT/ASCT, for example, in PFS time with a hazard ratio of 6.4 (*p* = 0.002) and in OS time with a hazard ratio of 8 (*p* = 0.004) (Table 4).

Myeloma patients over 58 years of age had a hazard ratio of 1.8 for inferior OS time. Myeloma patients with high initial disease stage had a hazard ratio of 1.7 for inferior PFS time. Cytogenetic risk was not associated with treatment outcome. Our data suggest that *PPM1D* gene mutation may be a predictor of inferior survival in myeloma patients treated with HDCT/ASCT independent of age, ISS and cytogenetic risk.

## 4. Discussion

In the general population, the prevalence of *PPM1D* gene mutations in peripheral blood cells has been reported in the context of clonal hematopoiesis (CH) at 0.13–5% [12,13], with a higher prevalence of somatic mutations in people of older age [23], while in patients exposed to chemotherapy, the prevalence of *PPM1D* gene mutations has been reported at 2–20%. In lymphoma patients, after several lines of chemotherapy and CAR-T cell therapy, the prevalence of *PPM1D* gene mutations was 20% and there was an association with inferior treatment outcomes [24]. Here, we investigated the prevalence of *PPM1D* gene mutations in the peripheral blood of myeloma patients who had been treated with chemotherapy, HDCT and ASCT. In a cohort of 75 myeloma patients, we identified four gDNA samples with low-frequency *PPM1D* gene mutations, indicating a 5% prevalence. A prevalence of 4% *PPM1D* gene mutations was previously reported in a targeted sequencing study on the stem cell products of myeloma patients treated by ASCT at the Dana-Farber Cancer Institute [17]. In our study, we sequenced the gDNA of peripheral blood cells collected after first and second ASCT, allowing the estimation of differential mutation prevalence. After several chemotherapy lines, HDCT and ASCT, the prevalence of truncating *PPM1D* gene mutations in the peripheral blood of myeloma patients emerged at 1.3% after the first HDCT/ASCT and 7.3% after the second HDCT/ASCT procedure. The rising prevalence of *PPM1D* gene mutations after second ASCT is reminiscent of the rising prevalence of other CHIP gene mutations in myeloma patients after ASCT. According to the MMRF COMMPASS study, the CHIP mutation prevalence rises from 5.8% at MM diagnosis to 25% three years after ASCT. Nevertheless, the prevalence of *PPM1D* gene mutations varies in lymphoma and myeloma after HDCT/ASCT with lower prevalence in myeloma. HDCT consists of genotoxic alkylating agents. The anti-cancer activity of melphalan chemotherapy derives from the alkylation of the DNA nucleotide, guanine, which creates crosslinks between DNA strands [25]. High-dose melphalan exposure increases both the overall and nonsynonymous mutational burden detected between myeloma diagnosis and relapse by ~10–20% [9]. The mutations acquired after melphalan HDCT are typically associated with DNA damage [26]. WIP1 phosphatase encoded by the *PPM1D* gene targets the tumor suppressor protein p53 and other proteins involved in the DNA damage response (DDR) [12]. Increased mutational load may increase the clonal complexity in myeloma without affecting the treatment outcome [26]. The side effects of HDCT treatment include tissue toxicity and secondary malignancies, which may be more prevalent in tandem HDCT settings [27]. If *PPM1D* gene mutations were caused by exposure to alkylating agents, the prevalence should be similar in all ASCT recipients including lymphoma and myeloma patients. Differences in the evolution of lymphoma and myeloma may cause the differential prevalence of *PPM1D* gene mutations in the two B-cell malignancies.

The ASCT parameters in the *PPM1D*-mutated (*PPM1D*mut) vs. *PPM1D* wild-type (*PPM1D*wt) subset of myeloma patients were comparable, with normal stem cell mobilization and stem cell yields before ASCT, but there was a larger amount of platelet transfusions required in patients with *PPM1D* gene mutations, indicating impaired regeneration in these ASCT recipients. This is reminiscent of findings in a larger cohort study, where patients with *DNMT3A* and *PPM1D* single and co-mutated CH had reduced stem cell yields and delayed platelet count recovery following ASCT [18].

The occurrence of low-frequency PPM1D gene mutations in the context of CH may predict inferior outcomes after ASCT in myeloma patients. In the studied cohort, the *PPM1D*-mutated (*PPM1D*mut) subset of myeloma patients had inferior treatment outcomes, with median progression-free survival (PFS) of 15 vs. 37 months and median overall survival (OS) of 36 vs. 156 months. Low-frequency *PPM1D* gene mutation may be causing inferior outcomes in myeloma with chromosome 1q gain, as there was an association with *PPM1D* gene mutations in this context. Extra copies of chromosome 1q21 have been associated with worse outcomes in multiple myeloma [28]. In our study, we observed a similar median PFS time and a shorter median OS time in patients with chromosome 1q gain compared to normal chromosome 1. Myeloma subclones with chromosome 1q gain express a specific transcriptomic signature and frequently expand during different treatments [29,30]. Moreover, these subclones shape an immune-suppressive environment by the upregulation of inflammatory cytokines and close interaction with the myeloid compartment. This environment may promote the emergence of *PPM1D* gene mutations or the survival of *PPM1D* mutated myeloma cells. To confirm the impact of low-frequency *PPM1D* gene mutations on treatment outcome and to investigate the potential association with chromosomal aberrations, a larger study in myeloma patients treated with HDCT and ASCT will be required.

## Figures and Tables

**Table 1:** *PPM1D* gene mutations in peripheral blood of MM patients after HDCT/ASCT.

Classification	Locus Chr7	VAF	NT Change	AA Change
indel	60,663,137	0.014	CA/C	D470fs *
indel	60,663,171	0.011	TA/T	A481fs *
nonsense	60,663,262	0.011	C/T	Q510 *
nonsense	60,663,388	0.016	C/T	R552 *
missense	60,663,461	0.051	T/G	V576G
missense	60,663,467	0.025	T/G	L578R

Abbreviations: indel: insertion/deletion; VAF: variant allele frequency. NT: nucleotide; AA: amino acid. * (asterisk) = translation termination (stop) codon.

**Table 2:** Clinical characteristics of myeloma patients at first diagnosis, univariate analysis.

	All Patients (*n* = 75)	*PPM1D*wt (*n* = 71)	*PPM1D*mut (*n* = 4)	*p*-Value
Sex				0.58
Female, *n* (%)	23 (31%)	21 (30%)	2 (50%)	
Male, *n* (%)	52 (69%)	50 (70%)	2 (50%)	
Age at ID, median (range)	59 (39–74)	60 (39–74)	55 (46–65)	0.40
Initial Disease Stage (ISS)				0.67
I	25 (33%)	24 (34%)	1 (25%)	
II	29 (39%)	28 (39%)	1 (25%)	
III	21 (28%)	19 (27%)	2 (50%)	
Cytogenetic risk category	*n* = 66	*n* = 62	*n* = 4	0.99
high risk *	18 (27%)	17 (27%)	1 (25%)	
standard risk	48 (73%)	45 (73%)	3 (75%)	
Cytogenetic aberrations	*n* = 66	*n* = 62	*n* = 4	
−13	12 (21%)	10 (19%)	2 (50%)	0.19
−14	5 (9%)	4 (8%)	1 (25%)	0.31
−16	4 (7%)	3 (6%)	1 (25%)	0.26
+1q	18 (32%)	15 (29%)	3 (75%)	0.09
del17p *	6 (11%)	6 (12%)	0	0.99
t(4;14) *	9 (16%)	8 (15%)	1 (25%)	0.51
t(14;16) *	3 (5%)	3 (6%)	0	0.99
t(11;14)	9 (16%)	8 (15%)	1 (25%)	0.51
Paraprotein-type				
Heavy chain IgG	41 (55%)	40 (57%)	1 (25%)	0.32
Heavy chain IgA	16 (22%)	13 (19%)	3 (75%)	0.03
Light chain only	17 (23%)	17 (24%)	0	0.57
Light chain kappa	54 (73%)	52 (74%)	2 (50%)	0.29
Light chain lambda	20 (27%)	18 (26%)	2 (50%)	0.29
Anemia				
Hb (g/L), average (range)	109 (46–167)	109 (46–167)	113 (92–128)	0.76
Hb < 110 g/L	34 (49%)	32 (48%)	2 (50%)	
Hb > 110 g/L	36 (51%)	34 (51%)	2 (50%)	
Hypercalcemia				
Ca (mmol/L), median (range)	2.4 (2–4.2)	2.4 (2–4.2)	2.5 (2.3–4.1)	0.55
>2.6 mmol/L	16 (23%)	16 (24%)	0	
<2.6 mmol/L	55 (77%)	51 (76%)	4 (100%)	
Beta-2-microglobulin				
B2M (mg/L), median (range)	3.4 (1–38)	3.4 (1–38)	3.9 (2–15)	0.58
>3.5 mg/L	35 (47%)	33 (46%)	2 (50%)	
<3.5 mg/L	40 (53%)	38 (54%)	2 (50%)	
Lactate-dehydrogenase				
LDH (U/L), median (range)	336 (110–3277)	342 (110–3277)	251 (154–428)	0.41
>480	9 (13%)	9 (14%)	0	
<480	59 (87%)	55 (86%)	4 (100%)	
Serum albumin				
g/dL, median (range)	3.5 (1.8–5.1)	3.5 (1.8–5.1)	3.6 (3.3–4.0)	0.72
>3.5 g/dL	32 (47%)	30 (47%)	2 (50%)	
<3.5 g/dL	36 (53%)	34 (53%)	2 (50%)	
Bone marrow infiltration				
Percent, median (range)	70 (3–100)	70 (3–100)	55 (40–100)	0.94
3–39%	16 (23%)	16 (24%)	0	
40–100%	55 (77%)	51 (76%)	4 (100%)	
Osteolytic lesions	57 (76%)	53 (75%)	4 (100%)	0.57
Renal dysfunction	15 (21%)	14 (21%)	1 (25%)	0.99

* High-risk cytogenetics are indicated for del17p, t(4;14) and t(14;16).

**Table 3:** Therapies and treatment responses in myeloma patients, univariate analysis.

	All Patients (*n* = 75)	*PPM1D*wt (*n* = 71)	*PPM1D*mut (*n* = 4)	*p*-Value
1st line therapy (ICT)				0.99
VD	13 (16%)	13 (17%)	0	
VRD	35 (47%)	33 (47%)	2 (50%)	
VCD	28 (37%)	26 (36%)	2 (50%)	
Response to 1st line ICT				0.99
CR	12 (16%)	11 (16%)	1 (25%)	
VGPR/PR	56 (75%)	53 (75%)	3 (75%)	
SD/PD	5 (7%)	5 (7%)	0	
not reported	2 (3%)	2 (3%)	0	
Response to 2nd line ICT	*n* = 52	*n* = 48	*n* = 4	0.99
CR	8 (15%)	8 (17%)	0	
VGPR/PR	27 (52%)	24 (50%)	3 (75%)	
SD/PD	9 (17%)	8 (17%)	1 (25%)	
not reported	8 (15%)	8 (17%)	0	
ICT lines				
n, median (range)	2 (1–7)	2 (1–7)	3 (2–6)	0.31
1–2	47 (63%)	46 (65%)	2 (50%)	
3–7	28 (37%)	26 (35%)	2 (50%)	
Relapse/Progression				
n, median (range)	1 (0–7)	1 (0–7)	2 (1–6)	0.26
0	21 (28%)	21 (30%)	0	
1	26 (35%)	25 (35%)	1 (25%)	
2–7	28 (37%)	26 (35%)	3 (75%)	
1st PBSC mobilization				
CD34+ cells (×10^6^/kg) (range)	10.7 (1–28)	10.7 (1–28)	10.6 (10–11)	0.96
<4 × 10^6^/kg	3 (4%)	3 (4%)	0	
1st HDCT				0.08
M	51 (68%)	49 (68%)	2 (50%)	
MB	8 (11%)	6 (9%)	2 (50%)	
MT	16 (21%)	16 (23%)	0	
1st ASCT				
CD34+ cells (×10^6^/kg) (range)	3.8 (1.3–9.9)	3.8 (1.3–9.9)	3.4 (2.9–3.9)	0.53
<2.5 × 10^6^/kg	4 (5%)	4 (5%)	0	
Platelet transfusions n, median (range)	3 (0–45)	3 (0–45)	9 (2–27)	0.15
Response to 1st ASCT				0.99
CR/sCR	48 (64%)	45 (63%)	3 (75%)	
PR/VGPR	22 (29%)	21 (29%)	1 (25%)	
SD/PD	1 (1%)	1 (1%)	0	
not reported	4 (5%)	4 (6%)	0	
2nd ASCT	*n* = 41	*n* = 37	*n* = 4	0.93
CD34+ cells (×10^6^/kg) (range)	3.8 (0.1–10)	3.8 (0.1–10)	3.7 (3.4–3.9)	
<2.5 × 10^6^/kg	3 (7%)	3 (8%)	0	
Response to 2nd ASCT	*n* = 41	*n* = 37	*n* = 4	0.17
CR/sCR	33 (75%)	31 (84%)	2 (50%)	
VGPR/PR	7 (17%)	5 (14%)	2 (50%)	
SD/PD	1 (3%)	1 (2%)	0	
HDCT/ASCT lines				
n, median (range)	2 (1–4)	2 (1–4)	2.5 (2–3)	0.01
1	34 (45%)	34 (47%)	0	
2	34 (45%)	32 (46%)	2 (50%)	
3	6 (8%)	4 (6%)	2 (50%)	
4	1 (1%)	1 (1%)	0	
Radiotherapy	28 (37%)	26 (37%)	2 (50%)	0.63

Abbreviations: immuno-chemotherapy (ICT); velcade and dexamethasone (VD); velcade, revlimid and dexamethasone (VRD); velcade, cyclophosphamide and dexamethasone (VCD); melphalan (M); melphalan and bendamustine (MB); melphalan and treosulfan (MT); complete remission (CR); partial remission (PR); stable disease (SD); progressive disease (PD).

**Table 4:** Clinical outcome, multivariate analysis.

	PFS		OS	
Predictors	HR (95% CI)	*p*-Value	HR (95% CI)	*p*-Value
*PPM1D*mut	6.42 (1.0, 20.7)	0.002	8.05 (2.0, 33.3)	0.004
Age > 58	1.30 (0.7, 2.4)	0.2	1.79 (0.7, 4.5)	0.2
ISS2/3 vs. ISS1	1.72 (0.9, 3.4)	0.12	1.32 (0.5, 3.5)	0.6
Cytogenetic high risk	0.98 (0.5, 2.1)	>0.9	0.93 (0.3, 3.2)	>0.9

HR hazard ratio, CI confidence interval.

## Data Availability

Data available on request due to restrictions, privacy and ethics.

## References

[1] Kyle R.A., Rajkumar S.V. (2008). Multiple Myeloma. Blood.

[2] Palumbo A., Anderson K. (2011). Multiple Myeloma. N. Engl. J. Med..

[3] Willenbacher E., Balog A., Willenbacher W. (2018). Short Overview on the Current Standard of Treatment in Newly Diagnosed Multiple Myeloma. Memo-Mag. Eur. Med. Oncol..

[4] Rajkumar S.V. (2011). Treatment of Multiple Myeloma. Nat. Rev. Clin. Oncol..

[5] Callander N.S., Silbermann R., Kaufman J.L., Godby K.N., Laubach J., Schmidt T.M., Sborov D.W., Medvedova E., Reeves B., Dhakal B. (2024). Daratumumab-Based Quadruplet Therapy for Transplant-Eligible Newly Diagnosed Multiple Myeloma with High Cytogenetic Risk. Blood Cancer J..

[6] Goldschmidt H., Ashcroft J., Szabo Z., Garderet L. (2019). Navigating the Treatment Landscape in Multiple Myeloma: Which Combinations to Use and When?. Ann. Hematol..

[7] Farag S., Bacher U., Jeker B., Legros M., Rhyner G., Lüthi J.-M., Schardt J., Zander T., Daskalakis M., Mansouri B. (2022). Adding Bendamustine to Melphalan before ASCT Improves CR Rate in Myeloma vs. Melphalan Alone: A Randomized Phase-2 Trial. Bone Marrow Transplant..

[8] Gillich C., Akhoundova D., Hayoz M., Aebi Y., Largiadèr C.R., Seipel K., Daskalakis M., Bacher U., Pabst T. (2023). Efficacy and Safety of High-Dose Chemotherapy with Treosulfan and Melphalan in Multiple Myeloma. Cancers.

[9] Maura F., Weinhold N., Diamond B., Kazandjian D., Rasche L., Morgan G., Landgren O. (2021). The Mutagenic Impact of Melphalan in Multiple Myeloma. Leukemia.

[10] Pich O., Reyes-Salazar I., Gonzalez-Perez A., Lopez-Bigas N. (2022). Discovering the Drivers of Clonal Hematopoiesis. Nat. Commun..

[11] Bernstein N., Spencer Chapman M., Nyamondo K., Chen Z., Williams N., Mitchell E., Campbell P.J., Cohen R.L., Nangalia J. (2024). Analysis of Somatic Mutations in Whole Blood from 200,618 Individuals Identifies Pervasive Positive Selection and Novel Drivers of Clonal Hematopoiesis. Nat. Genet..

[12] Husby S., Hjermind Justesen E., Grønbæk K. (2021). Protein Phosphatase, Mg^2+^/Mn^2+^-Dependent 1D (PPM1D) Mutations in Haematological Cancer. Br. J. Haematol..

[13] Kahn J.D., Miller P.G., Silver A.J., Sellar R.S., Bhatt S., Gibson C., McConkey M., Adams D., Mar B., Mertins P. (2018). PPM1D-Truncating Mutations Confer Resistance to Chemotherapy and Sensitivity to PPM1D Inhibition in Hematopoietic Cells. Blood.

[14] Kleiblova P., Shaltiel I.A., Benada J., Ševčík J., Pecháčková S., Pohlreich P., Voest E.E., Dundr P., Bartek J., Kleibl Z. (2013). Gain-of-Function Mutations of PPM1D/Wip1 Impair the P53-Dependent G1 Checkpoint. J. Cell Biol..

[15] Gibson C.J., Lindsley R.C., Tchekmedyian V., Mar B.G., Shi J., Jaiswal S., Bosworth A., Francisco L., He J., Bansal A. (2017). Clonal Hematopoiesis Associated With Adverse Outcomes After Autologous Stem-Cell Transplantation for Lymphoma. J. Clin. Oncol..

[16] Lackraj T., Ben Barouch S., Medeiros J.J.F., Pedersen S., Danesh A., Bakhtiari M., Hong M., Tong K., Joynt J., Arruda A. (2022). Clinical Significance of Clonal Hematopoiesis in the Setting of Autologous Stem Cell Transplantation for Lymphoma. Am. J. Hematol..

[17] Mouhieddine T.H., Sperling A.S., Redd R., Park J., Leventhal M., Gibson C.J., Manier S., Nassar A.H., Capelletti M., Huynh D. (2020). Clonal Hematopoiesis Is Associated with Adverse Outcomes in Multiple Myeloma Patients Undergoing Transplant. Nat. Commun..

[18] Stelmach P., Richter S., Sauer S., Fabre M.A., Gu M., Rohde C., Janssen M., Liebers N., Proynova R., Weinhold N. (2023). Clonal Hematopoiesis with DNMT3A and PPM1D Mutations Impairs Regeneration in Autologous Stem Cell Transplant Recipients. Haematologica.

[19] Maechler M., Bacher U., Daskalakis M., Nilius H., Nagler M., Taleghani B.M., Jeker B., Pabst T. (2023). Long-Term Safety of the Stem Cell Releasing Compound Plerixafor for Peripheral Stem Cell Collection in Myeloma Patients. Hematol. Oncol..

[20] Mehl J., Akhoundova D., Bacher U., Jeker B., Rhyner Agocs G., Ruefer A., Soltermann S., Soekler M., Winkler A., Daskalakis M. (2024). Daratumumab during Myeloma Induction Therapy Is Associated with Impaired Stem Cell Mobilization and Prolonged Post-Transplant Hematologic Recovery. Cancers.

[21] Duggan P., Reece D.E., Song K., Jimenez-Zepeda V., McCurdy A., Louzada M.L., Mian H.S., Sebag M., White D.J., Stakiw J. (2020). Is Tandem ASCT Needed in MM Patients with High Risk Cytogenetics in the Era of Maintenance Therapy? Results from the Canadian Myeloma Research Group (CMRG) Database. Blood.

[22] Talamo G., Dimaio C., Abbi K.K.S., Pandey M.K., Malysz J., Creer M.H., Zhu J., Mir M.A., Varlotto J.M. (2015). Current Role of Radiation Therapy for Multiple Myeloma. Front. Oncol..

[23] Zink F., Stacey S.N., Norddahl G.L., Frigge M.L., Magnusson O.T., Jonsdottir I., Thorgeirsson T.E., Sigurdsson A., Gudjonsson S.A., Gudmundsson J. (2017). Clonal Hematopoiesis, with and without Candidate Driver Mutations, Is Common in the Elderly. Blood.

[24] Seipel K., Frey M., Nilius H., Akhoundova D., Banz Y., Bacher U., Pabst T. (2023). Low-Frequency PPM1D Gene Mutations Affect Treatment Response to CD19-Targeted CAR T-Cell Therapy in Large B-Cell Lymphoma. Curr. Oncol..

[25] Poczta A., Rogalska A., Marczak A. (2021). Treatment of Multiple Myeloma and the Role of Melphalan in the Era of Modern Therapies—Current Research and Clinical Approaches. J. Clin. Med..

[26] Samur M.K., Roncador M., Aktas Samur A., Fulciniti M., Bazarbachi A.H., Szalat R., Shammas M.A., Sperling A.S., Richardson P.G., Magrangeas F. (2023). High-Dose Melphalan Treatment Significantly Increases Mutational Burden at Relapse in Multiple Myeloma. Blood.

[27] Lim H., Im M., Seo E.S., Cho H.W., Ju H.Y., Yoo K.H., Cho S.Y., Kim J.-W., Lim D.H., Sung K.W. (2024). Tandem High-Dose Chemotherapy Increases the Risk of Secondary Malignant Neoplasm in Pediatric Solid Tumors. Cancer Res. Treat..

[28] Neupane K., Fortuna G.G., Dahal R., Schmidt T., Fonseca R., Chakraborty R., Koehn K.A., Mohan M., Mian H., Costa L.J. (2024). Alterations in Chromosome 1q in Multiple Myeloma Randomized Clinical Trials: A Systematic Review. Blood Cancer J..

[29] Abdallah N., Greipp P., Kapoor P., Gertz M.A., Dispenzieri A., Baughn L.B., Lacy M.Q., Hayman S.R., Buadi F.K., Dingli D. (2020). Clinical Characteristics and Treatment Outcomes of Newly Diagnosed Multiple Myeloma with Chromosome 1q Abnormalities. Blood Adv..

[30] Tirier S.M., Mallm J.-P., Steiger S., Poos A.M., Awwad M.H.S., Giesen N., Casiraghi N., Susak H., Bauer K., Baumann A. (2021). Subclone-Specific Microenvironmental Impact and Drug Response in Refractory Multiple Myeloma Revealed by Single-Cell Transcriptomics. Nat. Commun..

